# Telehealth Transformation: A Mixed-Methods Study on Organizational Change Processes and Outcomes in a Private Medical Practice

**DOI:** 10.7759/cureus.57183

**Published:** 2024-03-29

**Authors:** Masab A Mansoor, Rashid Siddiqi

**Affiliations:** 1 Internal Medicine, Edward Via College of Osteopathic Medicine, Monroe, USA; 2 Cardiology, Methodist Health System, Houston, USA

**Keywords:** telemedicine, telemedicine experience, newer technology in healthcare, virtual care, telehealth

## Abstract

Background: This mixed-methods case study investigated the impacts, costs, barriers, and facilitators associated with implementing telehealth services across a private pediatric clinic system. The research examined the effects of telehealth on provider engagement and financial performance.

Methodology: Twenty-three clinicians, administrators, and staff across the pediatric clinics were interviewed before and after enterprise-level telehealth adoption to examine change processes amid this innovation. Twelve months of pre- and post-implementation financial records underwent statistical analysis to assess revenue and cost dynamics. Quantitative outcome measures encompassed expenses, revenues, and telehealth visit utilization rates, while qualitative analysis of interviews and focus groups revealed key implementation themes through rigorous inductive coding of participant narratives.

Results: Results showed significantly increased costs (44%) and revenues (47%) at clinics following virtual care expansion. Monthly telehealth visits per provider exponentially rose over 450%. Qualitatively, 83% of providers appreciated scheduling flexibility benefits, but 68% of staff cited workflow disruptions.

Conclusions: Interpretatively, findings demonstrated catalyzed financial and productivity transformations and nuanced perceived disruption amid pronounced appointment capacity expansions. Recommendations encompass updated care coordination protocols, enhanced training and support resources, incentivizing provider usage, and modulating implementation pacing responding to user feedback during large-scale organizational innovation.

## Introduction

Telehealth has experienced accelerated adoption in recent years, particularly during the COVID-19 pandemic, transforming healthcare delivery models [1]. However, significant knowledge gaps persist regarding the organizational change processes, financial impacts, and provider engagement outcomes associated with large-scale virtual care implementation, especially in pediatric care settings [2,3]. The study's target organization, a pediatric practice serving primarily low-income and underinsured families in central Louisiana, faces challenges such as transportation limitations and appointment delays that impede access to care [4]. To address these issues, the private pediatric practice is exploring the implementation of telehealth services.

This mixed-methods case study investigated the impacts, costs, barriers, and facilitators associated with implementing telehealth services across the organization's pediatric clinics. By examining the experiences of clinicians, administrators, and staff involved in the telehealth transition and analyzing shifts in revenues, expenses, and productivity metrics, the study sought to generate evidence-based insights to guide successful and sustainable virtual care integration in pediatric environments. The researchers hypothesized that implementing telehealth would increase both revenues and expenses. The research is grounded in Kotter's change management model and implementation science principles, considering factors such as leadership, resources, training, and stakeholder engagement [4,5]. Findings from this study can inform telehealth implementation strategies that improve access to high-quality pediatric care for underserved populations [6].

## Materials and methods

Study design and setting

This mixed-methods case study was conducted at a private pediatric organization with four central Louisiana clinics. The study employed a convergent parallel design, collecting and analyzing quantitative and qualitative data simultaneously to comprehensively understand the telehealth implementation process [7].

Participants and data collection

The study involved 23 participants, including clinicians, administrators, and staff across the pediatric clinics. Purposive sampling was used to select participants directly involved in the telehealth implementation process. Semi-structured interviews and focus groups were conducted before and after telehealth adoption to gather qualitative data on experiences, perceptions, and organizational change processes. Twelve months of pre- and post-implementation financial records were collected to assess revenue and cost dynamics.

Data analysis

Qualitative data from interviews and focus groups were transcribed verbatim and analyzed using thematic analysis [7,8]. NVivo software (QSR International, Melbourne, Australia) [9] was used to facilitate the coding process, and themes were inductively derived from participant narratives. Quantitative financial data were analyzed using descriptive statistics, paired-sample t-tests, and repeated measures analysis of variance (ANOVA) to compare pre- and post-implementation metrics, including expenses, revenues, and telehealth visit utilization rates. Statistical analyses were performed using the Statistical Package for the Social Sciences (IBM SPSS Statistics for Windows, IBM Corp., Version 26.0, Armonk, NY) [10].

Ethical considerations

The study was approved by the Ethics Committee of Mansoor Pediatrics with approval number 141301. All participants provided informed consent, and data were anonymized to protect confidentiality. Several stringent procedures were followed throughout the study methodology to avoid researcher bias and ensure objective, ethical interpretations of participant experiences with telehealth implementation. A reflexive journal enabled continually acknowledging and bracketing emergent subjective viewpoints or preconceptions requiring examination through memo writing [11]. Consultations with impartial peer debriefers fostered the facilitation of critical questioning of analytical decisions rather than relying solely on internal assessment. Intensive collaboration during the qualitative coding process allowed for resolving any discrepancies in theme derivation through consensus [12]. Member-checking procedures help verify that the realities portrayed in findings authentically resonate with participants’ perspectives [12,13]. Adhering to rigorous qualitative standards and striving for empathetic neutrality assists in bolstering credibility that the inevitable subjectivity innate to human research was adequately acknowledged and addressed.

## Results

Participant characteristics

The study involved 23 participants: five physicians, seven nurse practitioners, and 11 clinical staff members. Participants' years of experience ranged from two to 25 years.

Quantitative results

The financial metrics in Table 1 demonstrate the statistically significant impacts of incorporating telehealth services at the pediatric practice. A paired samples t-test revealed that following virtual care adoption, mean monthly revenues rose 46.81% at the organization's pediatric clinics (from $274,134 to $402,469). In comparison, expenses increased to 44.03% (from $211,404 to $304,491), with both changes highly significant (p<.001). Repeated measures ANOVA supported this finding, indicating a significant time effect on costs and revenues before versus after telehealth implementation, with follow-up tests showing the increases emerged immediately after adoption. Telehealth visits per provider exponentially grew by over 450%. Correlation analysis confirmed a robust positive relationship (r=.79) between providers’ telehealth utilization rates and overall clinic revenue.

**Table 1:** Means, Standard Deviations, and Paired-Samples T-test and Repeated ANOVA Analyses of Financial Metrics, Pre- and Post-telehealth Adoption ^a^ Revenues and expenses are reported in United States dollars. ^b ^Pre-adoption and post-adoption refer to the 12-months pre-adoption and 12-months post-adoption. ^*^p < .001; ANOVA: analysis of variance

Financial Metric^a^	Pre-adoption^b ^mean	Pre-adoption^b ^standard deviation	Post-adoption^b ^mean	Post-adoption^b ^standard deviation	Δ	t(11)^*^	F(5, 66)^*^
Monthly revenues	274,134.25	18,100.79	402,469.25	11,251.90	+46.81%	11.59	348.33
Monthly expenses	211,403.83	13,827.61	304,490.92	1,800.15	+44.03%	-10.23	191.44
Telehealth visits per provider	2.42	1.16	13.66	2.53	+464.5%	-19.04	232.43

Incorporating telehealth drove substantially higher costs, greater clinical productivity, and dramatic revenue gains once appointment capacities expanded. Statistical tests uniformly rejected null hypotheses of no financial impacts post-implementation. Further qualitative research can reveal the nuances behind these financial trends. However, quantitative data demonstrates the cost and revenue transformations and exponential appointment growth catalyzed by telehealth adoption.

Qualitative results

Thematic analysis of interviews and focus groups revealed two main themes: improved access and disruption to established workflows.

Improved Access and Convenience

Seventeen participants (73%) reported that telehealth adoption increased perceived convenience and appointment availability, facilitating access for patients facing geographic and transportation barriers. Providers highlighted the ability to accommodate higher daily patient volumes while maintaining visit quality. Reduced travel burdens and flexible scheduling were cited as key benefits for families.

Disruption to Established Workflows

Ten participants (43%) reported significant disruptions to established workflows during the telehealth transition. Challenges included integrating virtual care coordination, documentation difficulties, and confusion between care coordination between virtual and in-person visits. Participants emphasized the need for updated protocols, training, and technological enhancements to optimize telehealth integration.

## Discussion

This mixed-methods case study investigated the impacts, costs, barriers, and facilitators associated with implementing telehealth services at the pediatric organization. The findings demonstrate significant financial and operational transformations and perceived benefits and challenges experienced by providers and staff during the transition to virtual care.

The quantitative results revealed substantial increases in costs and revenues following telehealth adoption, aligning with previous research on the financial impacts of virtual care implementation [11]. The exponential growth in telehealth visits per provider suggests enhanced productivity and capacity, which may contribute to the observed revenue gains. However, the simultaneous rise in expenses highlights the need for careful cost management and resource allocation during telehealth integration [14].

Qualitative findings shed light on the perceived benefits and challenges of telehealth adoption. Improved access and convenience emerged as critical advantages, with participants reporting increased appointment availability, reduced travel burdens for families, and flexible scheduling options. These findings corroborate existing literature on telehealth's potential to enhance access to care, particularly for underserved populations [14-19].

However, the study also revealed significant disruptions to established workflows during the telehealth transition. There were prominent themes related to integrating virtual care coordination, documentation difficulties, and care coordination confusion between virtual and in-office visits. These findings align with previous research highlighting the importance of addressing workflow integration, training, and technological enhancements to optimize telehealth implementation [17-19].

The study's findings have implications for pediatric practices seeking to adopt telehealth services. The financial data suggest that while telehealth can drive revenue growth, organizations must be prepared for increased costs associated with technology investments, training, and workflow adaptations. The qualitative themes underscore the need for comprehensive change management strategies, including updated protocols, staff training, and continuous quality improvement efforts to streamline virtual care integration [1,15].

This study's limitations include its focus on a single pediatric clinic system and relatively small sample size. Future research should explore telehealth implementation across diverse pediatric settings and employ larger samples to enhance generalizability. Longitudinal studies examining telehealth adoption's long-term financial and operational impacts would provide valuable insights.

## Conclusions

This mixed-methods case study provides insights into the complex process of implementing telehealth services in a pediatric clinic system. The findings demonstrate the potential for telehealth to improve access and convenience for patients while highlighting the significant financial and operational transformations involved in the adoption process. The study underscores the importance of addressing workflow disruptions, documentation challenges, and care coordination issues through comprehensive change management strategies. The insights gained can inform telehealth implementation efforts in other pediatric settings, emphasizing the need for careful planning, resource allocation, and ongoing support. Future research should explore telehealth adoption across diverse pediatric contexts to enhance generalizability and examine long-term impacts on financial sustainability, patient outcomes, and provider satisfaction. By leveraging the findings from this study, pediatric practices can navigate the telehealth landscape more effectively, ultimately improving access to high-quality care for the children and families they serve.
